# Medical and Philosophical Intelligence

**Published:** 1814-03

**Authors:** 


### MEDICAL AND PHILOSOPHICAL INTELLIGENCE.

ROYAL SOCIETY.—January 20th.—Sir H. Davy communicated, in a Letter to the President, a long paper from Paris, on a new gas discovered in that city by M. COURTOIS, a manufacturer of saltpetre. It appears that this gas was discovered above two years ago; but such is the deplorable state of scientific men in France, that no account of it was published till the arrival of our English philosopher there. M. Courtois communicated his discovery to Clement and Desormes, who made some experiments with the gas, and latterly M. Gay Lusac has devoted his attention to an examination of its history and properties. Mean time Sir Humphry has made a great number of experiments on it, and would have made several more had he not wanted the necessary apparatus in Paris. M. Courtois was led to the discovery by observing how rapidly his metal pots were corroded in preparing the different kinds of sea-weed, which he used for making carbonate of soda. When the soda is extracted from the sea-weed, the new gas is easily disengaged; disengaged; by pouring strong sulphuric acid on the residuum, at the temperature of 158°, a beautiful dense violet-coloured elastic fluid rises. This the French chemists propose calling *iode* gas, from ιωδης, *violaceous*; but Sir Humphry, considering the English idiom, denominates it *violaceous* gas. Its properties are singular: combined with hydrogen, with phosphorus, and with oxymuriate of silver, (argentane of Davy) it forms a peculiar acid; it is a simple or uncompounded gas, at a suitable temperature a permanently elastic fluid, but heavier than any known gas, 100 cubic inches weighing 95·5 grains: it is a non-conductor of electricity, experiences no change exposed tn the action of the Voltaic battery with charcoal, is not inflammable, and does not support combustion. As a simple substance it has many analogies with oxygen, chlorine, and the alkalies: like oxygen, it rapidly unites with the metals: mercury, tin, lead, zinc, and iron, are converted by it, in a moderate temperature, into salts of orange, yellow, and brown tints, which are soluble in spirits, ether, and water, and form beautiful pigments, and most probably may be equally serviceable in the dye-house. Exposed to a moderate cold, it condenses into solid plumbago-coloured crystals. Combined with hydrogen, it forms what the French call hydroionic gas. Like the alkalies, it unites with oxygen, from which it can be expelled by heat. The existence of this subtance confirms the opinion previously given by Sir Humphry, that acidity and alkalescence do not depend on any specific principle, but on certain modifications of matter. This chemist concludes his important paper with some observations on the necessity of a new nomenclature, and proposing several arbitrary terminations to distinguish the various substances which, according to the principles of a significant nomenclature, would be called *iodats*. But his observations on this head are, as usual, submitted with the utmost diffidence, and merely as hints on which some general principles may be founded. It is, indeed, evident that the whole nomenclatural theory of Lavoisier is completely set aside by the subsequent discovery of facts, and it is time that chemists would unite together to form a new chemical vocabulary better adapted to the actual state of our knowledge.

---

*Medical Society of London.*—The Anniversary Meeting of this Society will be held at their house, in Bolt Court, on Tuesday the 8th of March; when the officers and council for the ensuing year will be elected, and the names of the successful candidates for the medals announced. At half past three o'clock, precisely, the annual Oration will be delivered by Dr. George Rees, at the London Coffee House, Ludgate Hill; after which the Society will dine together, as usual.

---

The pressure of other matter prevented our inserting an account of Mr. Stevenson's introductory lecture on the eye and ear, delivered on the 25th of January, to a very respectable audience. The discourse commenced with some general observations on the admirable structure of the human body, and of the eye in particular; the utility of the senses was touched upon, and the "inestimable value of sight" was duly appreciated. The ingenious lecturer then took "a cursory and general survey of that mechanism by which the eye is so exactly adapted to the impressing agent, as to be capable of exhibiting exhibiting the phenomena of vision, and thereby of communicating to the soul so many diversified and animating perceptions." We were particularly pleased with the perspicuous description of the anatomy of the eye and the ear. The evident utility of these elegantly constructed and essential organs, was described in the most animated language. In the course of this lecture, Mr. Stevenson did not insist upon the necessity of separating these particular organs from the anatomy and diseases of other parts of the body, which are usually comprehended in the lectures on anatomy and surgery, by professors. He offered, however, some strong arguments in favour of such division, observing, that "the multiplicity of topics necessarily embraced in a general course, renders it impossible to enter into a minute description of parts which constitute so small a share of the vast and interesting whole. And for similar reasons a great majority of the diseases of these organs are slightly adverted to or unavoidably omitted." To this cause he attributed, in a great measure, "the lamentable prevalency of empyricism in the treatment of disorders of the eye and ear." We understand that Mr. Stevenson intends to repeat this course annually.

---

According to the report of several *Apothecaries*, residing in various districts of the metropolis, the following was the average of the diseases in London between December 20th and January 19th :—

| | | | |
|---|---|---|---|
| Anasarca | 18 | Dyspnœa | 44 |
| Ascites | 8 | Dyspepsia | 90 |
| Asthma | 182 | Dysuria | 7 |
| Acne | 3 | Ecthyma | 3 |
| Apoplexia | 13 | Eczema | 11 |
| Aphtha lactantium | 10 | Erysipelas | 28 |
| Amenorrhœa | 21 | Enteritis | 9 |
| Asthenia | 22 | Enterodynia | 8 |
| Abortio | 8 | Enuresis | 5 |
| Bronchitis acuta | 147 | Epilepsia | 7 |
| ——— chronica | 66 | Epistaxis | 3 |
| Cancer | 5 | Febris remittens | 58 |
| Chlorosis | 16 | ——— intermittens | 14 |
| Cholera | 6 | Gastritis | 1 |
| Chorea | 2 | Gastrodynia | 22 |
| Cephalalgia | 22 | Hæmatemesis | 1 |
| Colica | 28 | Hæmoptoe | 9 |
| Catarrhus | 312 | Hæmorrhois | 10 |
| Convulsio | 20 | Hepatitis | 35 |
| ——— Puerpera | 1 | Hysteria | 13 |
| Carditis | 2 | Hydrocephalus | 7 |
| Cynanche Tonsillaris | 56 | Hydrothorax | 3 |
| ——— maligna | 9 | Herpes | 18 |
| ——— Trachealis | 4 | Hypochondriasis | 12 |
| ——— parotidea | 10 | Icterus | 12 |
| Diabetes | 1 | Impetigo | 4 |
| Dysenteria | 13 | Ischuria | 2 |
| Diarrhœa | 82 | Leucorrhœa | 3 |

| | |
|---|---|
| Lepra | 2 |
| Lichen | 2 |
| Mania | 1 |
| Menorrhagia | 25 |
| Miliaria | 2 |
| Morbi Infantiles | 102 |
| ——— Biliosi | 89 |
| Nephritis | 4 |
| Nephralgia | 1 |
| Obstipatio | 3 |
| Ophthalmia | 42 |
| Ophthalmica | 1 |
| Paralysis | 13 |
| Phthisis Pulmonalis | 35 |
| Pertussis | 37 |
| Peritonitis | 3 |
| Phrenitis | 9 |
| Pneumonia | 119 |
| Podagra | 25 |
| Porrigo larvalis | 5 |
| ——— scutulata | 3 |
| ——— favosa | 2 |
| Psoriasis | 15 |
| Purpura | 2 |
| Pyrosis | 9 |
| Pericarditis | 1 |
| Rheumatismus acutus | 72 |
| Rheumatismus chronicus | 70 |
| Rubeola | 15 |
| Scabies | 91 |
| Scarlatina simplex | 5 |
| ——— anginosa | 15 |
| Splenitis | 1 |
| Scrofula | 30 |
| Synochus | 5 |
| Sycosis capillitii | 1 |
| Tabes Mesenterica | 9 |
| Trismus | 1 |
| Typhus | 12 |
| Tic Douloureux | 1 |
| Variola | 12 |
| Varicella | 17 |
| Vermes | 38 |
| Vertigo | 23 |
| Urticaria | 6 |
| Total | 2502 |

The Deaths in the Bills of Mortality from December 24th to January 28th, 1814, according to the returns of the *Parish Clerks*, were as under:—

| | |
|---|---|
| Males . . . . | 779 |
| Females . . . | 701 |
| Total | 1480 |

The following recipe is recommended as a most efficacious remedy for Chapped Nipples :—℞. Boracis, ʒſs. Mellis, ℥ſs. Farinæ tritics, q. s. ad consistentiem idoneam.—This is to be spread thickly on lint, and applied to the parts affected. Honey is in itself an excellent application for this purpose. Our readers will probably recollect, that two very useful and popular remedies are, honey and the salt prunella. If the latter be used, a piece of it is usually carried in the pocket, and after being moistened with the tongue, is smeared over the chaps several times a day, and a cure is soon effected. Whether the combination of this salt with honey be equally efficacious with the borax in relieving the affection of the nipples, must remain for future experiments to determine.

A contagious fever producing an alarming and daily increasing mortality having prevailed at Gibraltar during the summer and autumnal months of 1813, the following account of it, drawn up by a medical officer of that garrison, will be found interesting. After stating that on the 3d of December last the disease totally disappeared, the writer proceeds :

When the disease commenced, the population was 15,600 inhabitants; bitants; and the garrison with their families amounted to 5,500. Of their inhabitants, nearly one-half took shelter on board of ships, or were forced to encamp on the neutral ground, where they all continued well, with very few exceptions, none having been taken ill after they had quitted the place six days. Of the inhabitants who remained, to the number of 7,870, upwards of 3,800 had had the disease in 1804, who all escaped, no well-authenticated case having appeared of any person taking the fever a second time: of the remainder, not more than 40 escaped an attack of the fever; yet upwards of 2,600 of the garrison and their families escaped, by their being encamped outside of the town and on the heights above it, and avoiding all communication with the town.

The disease appeared to originate solely from contagion, as every person outside of the walls, or who kept themselves in complete seclusion, remained in perfect safety: and all the numerous vessels lying in the Mole and Bay, though crowded with inhabitants, continued perfectly healthy whilst they avoided communication with the town; but in six different instances, where they neglected this precaution, the fever appeared on board.

The average mortality of deaths was one in five. In more than half of the fatal cases, the black vomit took place. Yellowness of skin was rather an uncommon symptom, and seldom occurred but in fatal cases, and then was of a dingey mahogany colour, and commenced a few hours before death.

The faculty now seem generally to agree that the disease is similar to that of 1804, and that it is a distinct disease from the bilious remittent fever, and has been introduced by imported contagion, and propagated by contagion alone, as the season has been one of the coolest and healthiest ever known.

---

We have great pleasure to announce that Mr. Brooks, of Blenheim-street, has liberally opened to the medical profession, and men of science, his very curious and extensive museum; the result of many years of personal labour, intense mental application, and considerable expenditure of money.

The building which contains this great and choice collection of anatomical Preparations, human and comparative, with specimens illustrative of the most interesting classes of natural history, consists of a principal room, forty-five feet in length, twenty-one broad, and forty high; with two lateral apartments, opening from the large room, each eighteen feet in length, ten wide, and of a proportionate height. The whole is fitted up and decorated in the Egyptian style, executed with great truth after the designs, and under the direction of *Mr. Bond*, an architect eminent for his chaste and classic taste.

---

Mr. Armiger, has been unanimously elected the surgeon of the Eastern Dispensary.

---

*Third Report of the London Committee of Apothecaries and Surgeon-Apothecaries of England and Wales.*

The Resolutions of this Committee of Sept. 4, 1813, which were proposed and published as the bases of a new bill, were sent to each of the chartered medical bodies.

On Oct. 29th ult. the Court of Assistants of the Society of Apothecaries passed a resolution (No. 1), which was directed to Mr. Ward, Secretary to the Committee.

A note from Mr. Balfour, Secretary to the College of Surgeons, dated Oct. 11th ult. was also addressed to Mr. Ward, which merely acknowledged acknowledged that the Memorial, and the Resolutions of Sept. 4th, had been received.

The Committee conceived it to be their duty to direct their Report, and the Resolutions before specified, to such Members of Parliament as had evinced an interest in the intended measure. In consequence a communication took place between the Right Honorable George Rose and the Committee; and the result was transmitted by Mr. Rose to the Executives of the Royal Colleges of Physicians and Surgeons, as was also a Resolution of the Committee (No. 2) dated Nov. 19th ult.

At this juncture, the petition for leave to bring in a bill was presented to the Honorable House of Commons, by John Calcraft, Esq.

On the 2d instant the Chairman was honored with a note from Mr. Rose, inclosing a letter he had received from the President, with the Report of the Royal College of Physicians (No. 3.)

Upon this, the Committee ordered copies of the said letter and report to be sent to the Masters of the College of Surgeons and of the Society of Apothecaries, which they accompanied with a Resolution, dated the 7th instant, importing their readiness to confer with each of those bodies on the subject of the Report. The note (No. 4) has been since sent from the College of Surgeons.

The Court of Assistants of the Society of Apothecaries met on Thursday last, and appointed a Committee of their body to take into consideration these several papers; and a conference between the two Committees was to take place at Apothecaries' Hall, on Friday the 25th instant.

The Committee have embraced the earliest opportunity of laying these proceedings before the medical public, but beg leave to refrain from offering any comment while a measure of such importance is in the course of arrangement.

*Feb.* 22, 1814.

No. 1. *Apothecaries' Hall,* *Oct.* 29, 1813.

Resolved—"That this Court, after taking into consideration the memorial addressed to them from the Committee of the Society of Apothecaries and Surgeon-Apothecaries of England and Wales, together with the reply of the Royal College of Physicians enclosed to their memorial, are of opinion, that this Court cannot enter into measures for any improvement in pharmacy but in conjunction with that learned body. "S. Backler, Clerk."

*To Mr. Ward, Secretary, &c.*

No. 2.

Resolution of the General Committee.

Resolved—That this Committee determine not to apply for the formation of a fourth medical body, for the purpose of examinations, if the powers of the present chartered bodies can be so extended as to accomplish the objects which the printed Resolutions of the 4th of September embrace.

The Committee will therefore be happy to confer with the executives of the three chartered bodies, upon the basis of the above statement; and prior to that conference, would prepare a detail of such propositions as they conceive will be proper to form the heads of a bill. But, as they consider themselves to be deputed by, and acting for, the Apothecaries and Surgeon-Apothecaries of England and Wales, they must reserve to themselves a right to make such proposals known to their constituents, before they are considered as giving a final assent.

*Committee Room, Nov.* 19, 1813.

No.

No. 3.

LETTER of DR. LATHAM, President of the Royal College of Physicians, to the Right Honorable GEORGE ROSE.

SIR, 2, *Harley-street, Jan.* 27, 1814.

I have the honor of transmitting to you the Report of the Committee of the Royal College of Physicians, as laid before the College at the last Comitia, and am sincerely happy in the prospect afforded of matters being at last satisfactorily adjusted; for although the College did not agree with the Committee in one point, yet you will see it is a point not at all affecting the material provisions of the bill. I have the honor to be with great respect, Sir,

Your very obedient Servant,

(Copy) J. LATHAM.

*Jan.* 25th, 1814.

Copy of the Report of the Committee appointed to take into consideration the papers relative to the bill intended to be framed and brought into parliament by the London Committee of Apothecaries, addressed by the Right Honorable George Rose to the President of the Royal College of Physicians, and by him submitted to the last Comitia Majora held at the College.

The Committee having met in pursuance of the orders of the College, and having deliberated upon the papers referred to their attention, *unanimously* agreed to recommend the following Resolution to the adoption of the College.

Resolved,—That the President be empowered to inform the Right Honorable George Rose, that the Royal College of Physicians have no objection to the formation of a bill to be brought into parliament by the London Committee of Apothecaries, upon the bases of certain Resolutions published by the said Committee, and dated the 4th of September, 1813, provided the powers therein contained be vested in the Society of Apothecaries, as established by the charter of King James; and provided the bill, before it shall be brought into the House of Commons, be submitted to the consideration of the College of Physicians for their examination and approval.

This Report was received and approved by the College, with the exception of the 4th Resolution, viz.

"That no person acting, or having acted as full surgeon or apothecary in the army or navy, shall be liable to an examination, except as to his qualification in midwifery." The College requiring that all persons who have acted in any department of the army or navy as medical practitioners, should not thence derive any authority to practise, unless examined by the constituted bodies.

No. 4.

(Copy.) *Royal College of Surgeons, Feb.* 12, 1814.

SIR,—I am directed by the Master of this College, to acknowledge the receipt of your letter and enclosures of the 8th instant, and to return you his thanks for the same. I am, Sir,

Your most obedient Servant,

WM. T. WARD, Esq. EDM. BELFOUR, Sec.

---

Dr. SQUIRE will on Tuesday 22d begin a Course of Lectures on the Theory and Practice of Midwifery, and the Diseases of Women and Children.

---

Dr. BURNETT, late Physician and Inspector of Hospitals to the Mediterranean Fleet, has in the press a work, Part I. containing a practical Account of the Mediterranean Fever, with cases and dissections. Part II. the History of Fever in the Fleet during 1810, 1811, 1812, 1813; and of the Gibraltar and Carthagena Fever.

